# Sharp Disposal Practices in Urban Dispensaries

**DOI:** 10.4103/0970-0218.66866

**Published:** 2010-04

**Authors:** Sandeep Sachdeva, Utsuk Datta

**Affiliations:** Ministry of Health and Family Welfare, Nirman Bhawan, New Delhi-110 067, India. E-mail: drsandeepsachdeva@gmail.com; 1 Department of Education and Training, National Institute of Health and Family Welfare, Munirka, New Delhi-110 067, India

Sir,

Bio-medical waste means waste generated during the process of diagnosis, treatment and/or immunization of human beings or animals or in research activities pertaining thereto or in the production or testing of biological. Injections are among the most frequently used medical procedure with an estimated 12 to 16 billion injections administered each year worldwide. (1) According to IPEN study, 03-06 billion injections are administered annually in India. (2) A large majority of them are administered for curative purpose and rest for immunization. An injection is considered to be safe when it does no harm to the recipient, does not expose the health care worker to any risk and does not result in waste that is dangerous to the community. (3) Although sharps waste constitutes a small proportion of all health care waste, its inadequate management can cause direct negative impact on health personnel and community and in addition, pollute the environment.

A study was conducted to assess injection [immunization] waste disposal practices in urban dispensaries of Delhi, India. Primary data were collected by a single investigator using observation checklist based on Government of India guidelines (4) during the period October 2005 to March 2006. All [32] government dispensaries operational in a randomly selected municipal zone of Delhi, India were covered after obtaining permission from competent authorities. Owing to limited resources, only one visit was made to each health facility and best of five injections were observed to comment on disposal practices and method of terminal waste disposal from the dispensary was also observed and/or recorded. Results are thus presented according to health facility.

**Figure 1:**
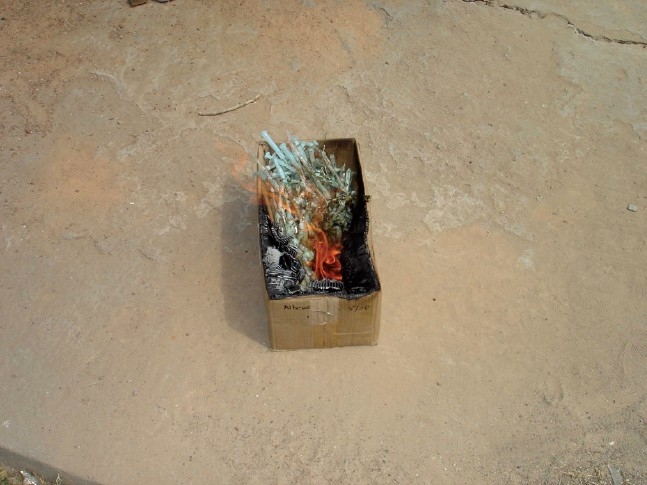
Uncontrolled burning of AD syringes in a dispensary

All the health facilities were using AD [auto-disable] syringes for primary immunization of children. Correct needle disposal practices [needle shredding/chemical disinfection] and incorrect practices [needle bent/recapped with both hands] were observed in equal number (16 [50.00%]) of health facilities respectively. It was noticed that dedicated bio-medical waste transport service was available in 18 [56.25%] health facilities only as per regulatory stipulation for transfer of biomedical including immunization waste to common biomedical treatment facility. Rest of the health facilities i.e. 14 (43.75%) were either disposing off their entire immunization waste into nearby general municipal waste containers [*dhalao*] or were burning in uncontrolled manner [Figure 1]. An interesting observation was made that incorrect needle practices were more prevalent in those health facilities that had provision of dedicated BMW transport service suggestive of carelessness/need for attitudinal change [Table 1].

**Table 1:** Sharp disposal practices according to health facilities

Used injection needle practices	Dedicated BMW transport service at health facility				Total	
	Available		Not-available		
	N	%	N	%	N	%
Correct	3	09.37	13	40.62	16	50.00
Incorrect	15	46.87	01	03.12	16	50.00

Total	18	56.25	14	43.75	32	[100]

BMW labeled colored coded bags were used in 29 [90.62%] health facilities for disposing immunization waste; however, varying color bags were in use. All the injection-related waste was also thrown in respective available bags. Red-colored bags were used in 16 [50.0%], blue bags in 11 [34.37%], and yellow bags in 2 [6.25%] health facilities, respectively. The reason cited was logistic issues. The cut-needle that was collected using hub cutter was emptied back directly into plastic/cardboard container at the end of immunization day or was being preserved in the hub cutter itself till it got completely filled. It was heartening to note that there was no littering of injection waste in or around any of the health facilities. IPEN study on ‘Assessment of Injection Practices in India’ reported satisfactory immunization waste disposal in 50.0% of health facilities only. There are multiple government health agencies in Delhi that are providing health services including immunization to community. Some of these agencies had outsource to vendors the practice of transporting biomedical including immunization waste to common biomedical treatment facility in accordance with Bio Medical Waste Management and Handling Rules, 1998 while in rest this was not made available.

To conclude, corrective interventions are required at two levels i.e. one at individual/health facility and another at health agency level. It is the organizational responsibility to coordinate and make available dedicated bio-medical waste transport services including other logistics, which is also binding on them as per statutory requirement. Re-orientation training/sensitization of health personnel at periodic interval may instill in safe BMW management practices. Similarly, close monitoring and supervision is required by medical officers at individual level to ensure correct waste disposal practices by health workers.
